# Near-field photochemical and radiation-induced chemical fabrication of nanopatterns of a self-assembled silane monolayer

**DOI:** 10.3762/bjnano.5.156

**Published:** 2014-09-03

**Authors:** Ulrich Christian Fischer, Carsten Hentschel, Florian Fontein, Linda Stegemann, Christiane Hoeppener, Harald Fuchs, Stefanie Hoeppener

**Affiliations:** 1 Physikalisches Institut, Interface Physics Group, Westfälische Wilhelms-University Münster, Wilhelm Klemm Str. 10, 48149 Münster, Germany; 2 Laboratory of Organic and Macromolecular Chemistry (IOMC) and Jena Center for Soft Matter (JCSM), Friedrich Schiller University, Humboldtstr. 10, 07743 Jena, Germany

**Keywords:** colloid lithography, contact lithography, near-field, photochemistry, self-assembled silane monolayers

## Abstract

A general concept for parallel near-field photochemical and radiation-induced chemical processes for the fabrication of nanopatterns of a self-assembled monolayer (SAM) of (3-aminopropyl)triethoxysilane (APTES) is explored with three different processes: 1) a near-field photochemical process by photochemical bleaching of a monomolecular layer of dye molecules chemically bound to an APTES SAM, 2) a chemical process induced by oxygen plasma etching as well as 3) a combined near-field UV-photochemical and ozone-induced chemical process, which is applied directly to an APTES SAM. All approaches employ a sandwich configuration of the surface-supported SAM, and a lithographic mask in form of gold nanostructures fabricated through colloidal sphere lithography (CL), which is either exposed to visible light, oxygen plasma or an UV–ozone atmosphere. The gold mask has the function to inhibit the photochemical reactions by highly localized near-field interactions between metal mask and SAM and to inhibit the radiation-induced chemical reactions by casting a highly localized shadow. The removal of the gold mask reveals the SAM nanopattern.

## Introduction

Chemical nanopatterns consist of spatially separated areas providing different chemically reactive groups. Hence, they form templates for the spatially defined fabrication of functionalities for the attachment of, e.g., biomolecules [1–3], polymers [4–6], or other organic or inorganic nanoparticles [7–8] with nanometer precision. Potential applications of such chemically structured templates are found in catalysis [9], biochemical surface engineering [10], cell adhesion [11], and biomineralization [12]. It has been shown previously that chemically nanostructured surfaces can be used as platforms for the graphoepitaxial growth of block-copolymer nanostructures [13] and to control the growth and mobility of cells on surfaces [14]. Top-down as well as bottom-up fabrication methods are applied in the field of chemical nanostructuring. Thus, chemical nanopatterns were formed by using different approaches, including electro-oxidative nanolithography [15], electron beam lithography [16], self-assembled block-copolymer structures [17], block-copolymer micelle nanolithography [18] as well as polymer blend lithography [19]. Moreover, gold nanoparticles with functional groups have been arranged in many different approaches to form chemical nanopatterns [14]. With parallel lithographic techniques based on self-assembly, such as colloidal sphere lithography (CL) [20–22], nanostructures extending over relatively large areas can be formed in a simple way. We demonstrated a new concept for the chemical fabrication of nanopatterns of self-assembled silane monolayers (SAM) with selective functional groups, in which metal nanostructures, fabricated by CL, served as replaceable barrier nanostructures to guide localized self-assembly processes [23]. Earlier, submicroscopic pattern replication with visible light was introduced [20,24] as a near-field photochemical process to generate chemical nanostructures. A sandwich between a metal nanostructure produced by CL and a monomolecular Langmuir–Blodgett layer of dye molecules was irradiated with light. Removing the metal mask revealed a chemical nanostructure consisting of regions of bleached and unbleached dye. The metal mask has an inhibitory function and protects the dye from photochemical bleaching by quenching the excited state of the dye molecules through energy transfer from the excited state to the metal [23]. In this near-field photochemical process it is the near field of the dipole-excited molecule, not the near field of the metal mask, which is important. This dipole-excitation is generated by far field illumination. The dipolar near field of the molecule leads to a quenching of the excited state of the molecule due to energy transfer to the metal nanostructure within the dipolar near field range of the molecule [25]. As a photochemical reaction is, in general, a chemical reaction starting from the excited state of the molecule, a quenching of the excited state competes with and inhibits the photochemical reaction. The short range of the dipolar near field of the excited molecule and thus of the energy transfer process leads to the potentially high resolution of the near-field photochemical process.

Here we explore an extension of this near-field concept by using metal masks as a strongly localized barrier for the destruction of a silane monolayer by chemical reactions induced by oxygen ions, reactive ozone, or by photochemical reactions through UV radiation or visible light. In this way we intend to create additional means for a more flexible formation of chemically functional nanostructures with the potential for a high spatial resolution in the range of 10 nm. Three fabrication processes are demonstrated. In process 1, a near-field photochemical process as described above is applied to bleach a SAM of dye molecules by exposure to visible light. Besides the utilization of photochemical effects, self-assembled monolayers can be structured by means of destructive irradiation and/or chemical activation with oxygen plasma or UV–ozone processing [26]. In process 2, oxygen-plasma induced nanostructuring, an oxygen plasma leads primarily to the chemical destruction of the amino groups of an (3-aminopropyl)triethoxysilane (APTES) SAM. For this process the close contact between mask and the very thin SAM substrate is essential in order to achieve a high resolution. But here the term near-field is not appropriate. The mask protects the substrate from the ion radiation. As ions impinge diffusively onto the sandwich structure from all directions, a well localized ion shadow exists for a very thin substrate only at a very short distance from the mask. On the other hand, this shadow casting function of the mask is not relevant for the near-field photochemical process described above, as it is not important from which side the light impinges on the sandwich structure of the metal mask and the SAM layer. Even for a highly transparent very thin metal mask the energy transfer process can be sufficient to quench the excited state of the molecules of the SAM and thus to inhibit the photochemical reaction [24–25]. These approaches are supplemented by process 3, UV light and ozone-induced nanostructuring, which may be regarded as a mixture of a radiation-induced chemical and a near-field photochemical process. In this case, the formation of the highly reactive ozone species and photochemical reactions result in a destruction of the amino groups of an APTES SAM. Common basis for all three structuring strategies is a metallic mask, which acts as a protection layer of the SAM. As a mask gold patterns were used, which were obtained through colloidal sphere lithography, a technique which takes advantage of the self-assembly of latex beads into hexagonally ordered monolayers on smooth surfaces, such as glass, mica, and silicon [20–22]. The evaporation of a metal layer and the subsequent removal of the spheres form the surface leads to a projection pattern in form of hexagonally arranged triangular-shaped metal islands on the substrate. The size and spacing of the triangular islands can be adjusted by the size of the latex beads. Different masks were utilized for process 1, and processes 2 and 3, respectively.

## Results

**Figure 1:**
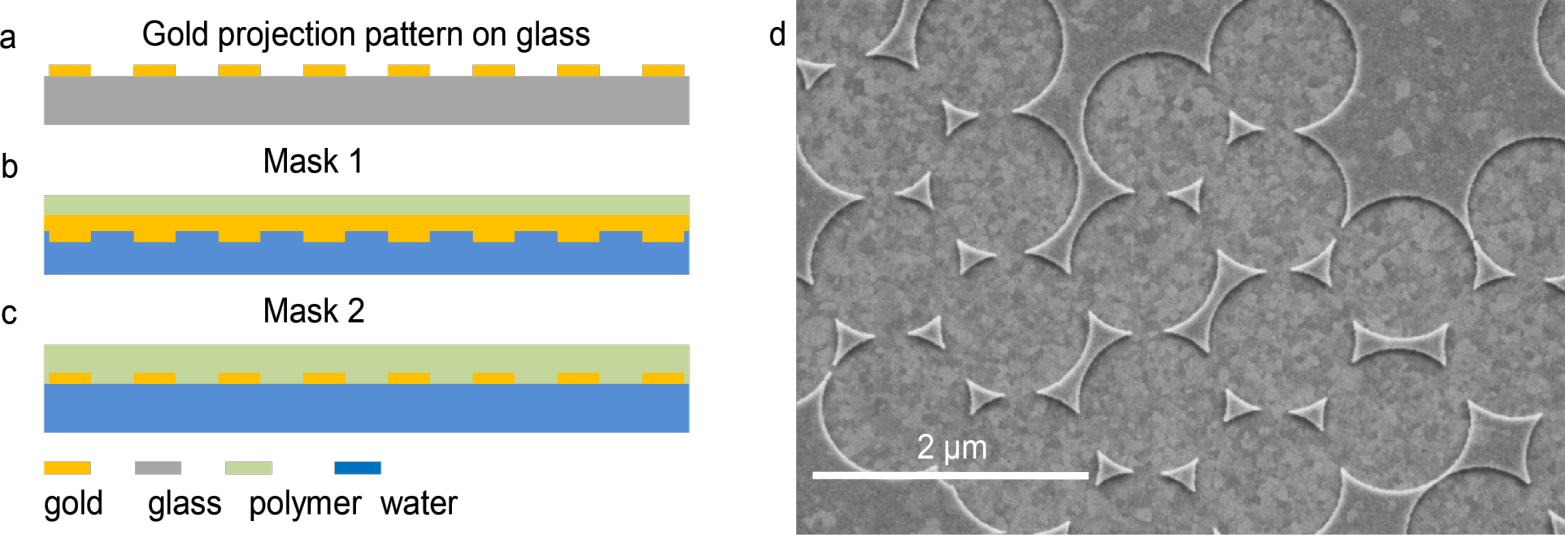
Schematic representation of the mask fabrication processes. (a) Colloidal lithographic gold projection patterns on glass. (b) Mask 1, supported by a metal film, floating on water ready for use. (c) Metal mask 2, embedded in polystyrene matrix, floating on water. (d) Scanning electron micrograph of mask 1, a metal-supported CL projection pattern.

In process 1 the mask consists of a 30 nm thick gold projection pattern that is supported by a 100 nm thick gold film (in the following referred to as mask 1). The main fabrication steps for the formation of such masks are outlined in Figure 1.

To form mask 1, a thin gold film backed by a thin film of polystyrene is transferred onto a gold projection pattern as obtained by CL. The projection pattern backed by the gold film is then transferred to a water surface. Mask 1 is then ready to be transferred to a SAM of dye molecules where it can be used for the contact imaging process 1 (for further details see Experimental section). Figure 1d shows a typical SEM micrograph of mask 1. Mask 2 for the processes 2 and 3 consists of 30 nm thick gold projection patterns obtained by CL with 1.2 µm or 0.22 µm latex beads. Mask 2 is supported by a flexible polymer matrix and transferred to a water surface. It is then ready to be transferred to an APTES-functionalized solid surface. Before exposure in process 2 and process 3 the supporting polymer film is removed.

**Figure 2:**
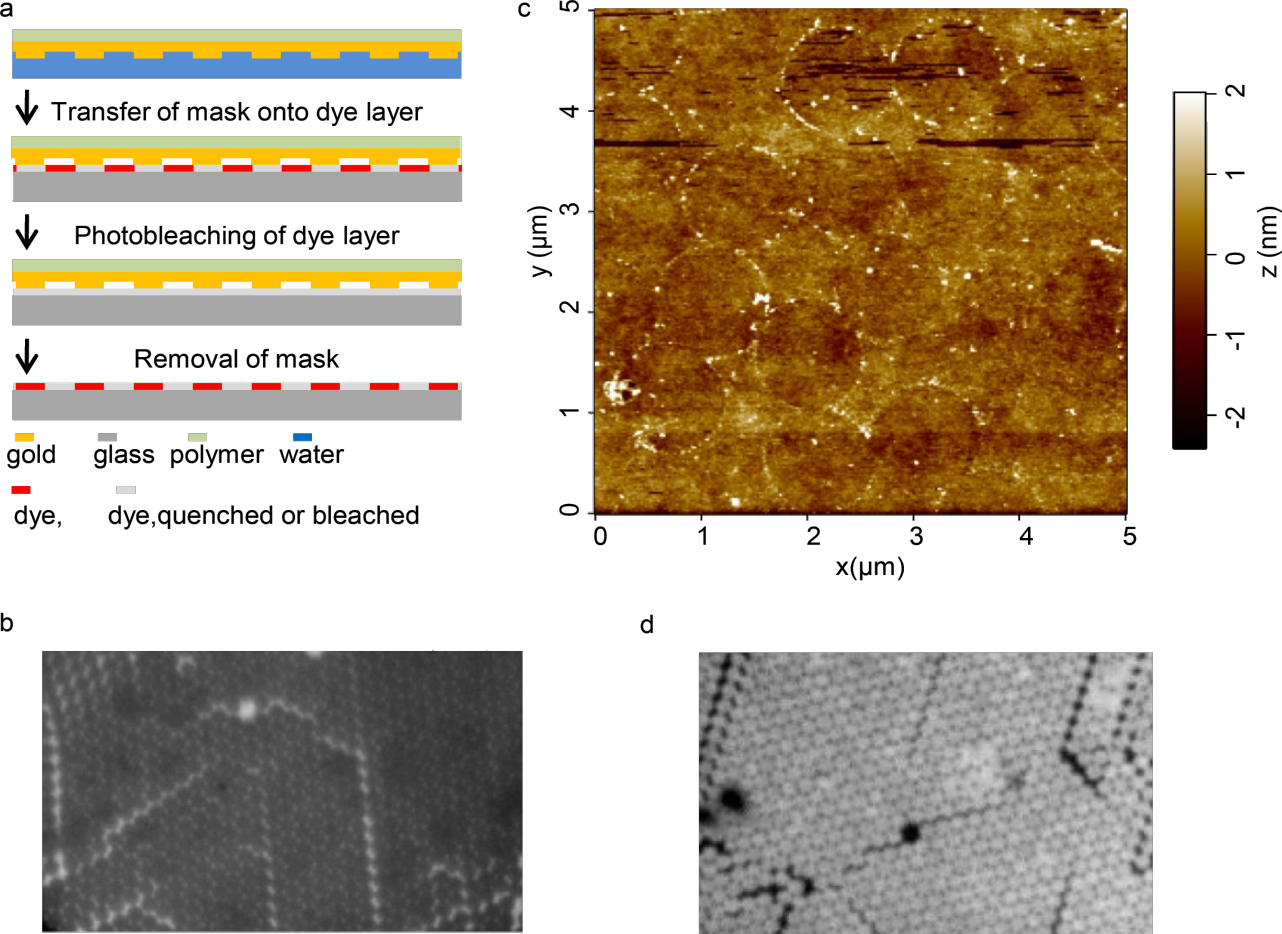
Photochemical near-field lithographic process 1: (a) Schematic outline of the steps involved in this process. Mask 1 is transferred to a fluorescent SAM. Light exposure leads to photobleaching of the unprotected areas. Removal of the mask after photobleaching releases the remaining molecules from the quenched state. (b) Epi-fluorescence image of a fluorescein monolayer after bleaching and removing the mask 1. (c) AFM topography image of the photochemical modification of the fluoresceine monolayer reveals a small topographic step from bleached to unbleached regions. (d) Epi-fluorescence image of a fluoresceine monolayer in close contact with mask 2.

A fluorescein self-assembled monolayer is used as a patternable substrate for process 1, which is outlined schematically in Figure 2a (for details on the preparation of the dye functionalized SAM see the Experimental section).

A sandwich of mask 1 and the fluorescein monolayer was subjected over an area of about 1 mm^2^ to the photochemical structuring process by irradiation with light of an appropriate wavelength. Afterwards the mask was removed with a piece of Scotch tape. Figure 2b shows a fluorescent image of the result of the process when mask 1 was removed after irradiation of the sandwich structure for 1 h (see Experimental section for details of the exposure conditions). A fluorescent copy of mask 1 was obtained. For the 1.2 µm projection pattern the typical triangular fluorescent patches are resolved. The reason for the fluorescence preservation is mainly the energy transfer from the dye molecules to the gold at sites of close contact with the mask, which effectively quenches the fluorescence and suppresses the bleaching processes [24–25]. Figure 2c shows the topography of such a fluorescent pattern as obtained with an atomic force microscope (AFM). The unbleached regions show a weak topographic step of the order of 1 nm with sharp edges from bleached to unbleached regions. The edge sharpness of the topographic pattern in this micrograph is limited by the pixel resolution of the AFM of about 30 nm.

**Figure 3:**
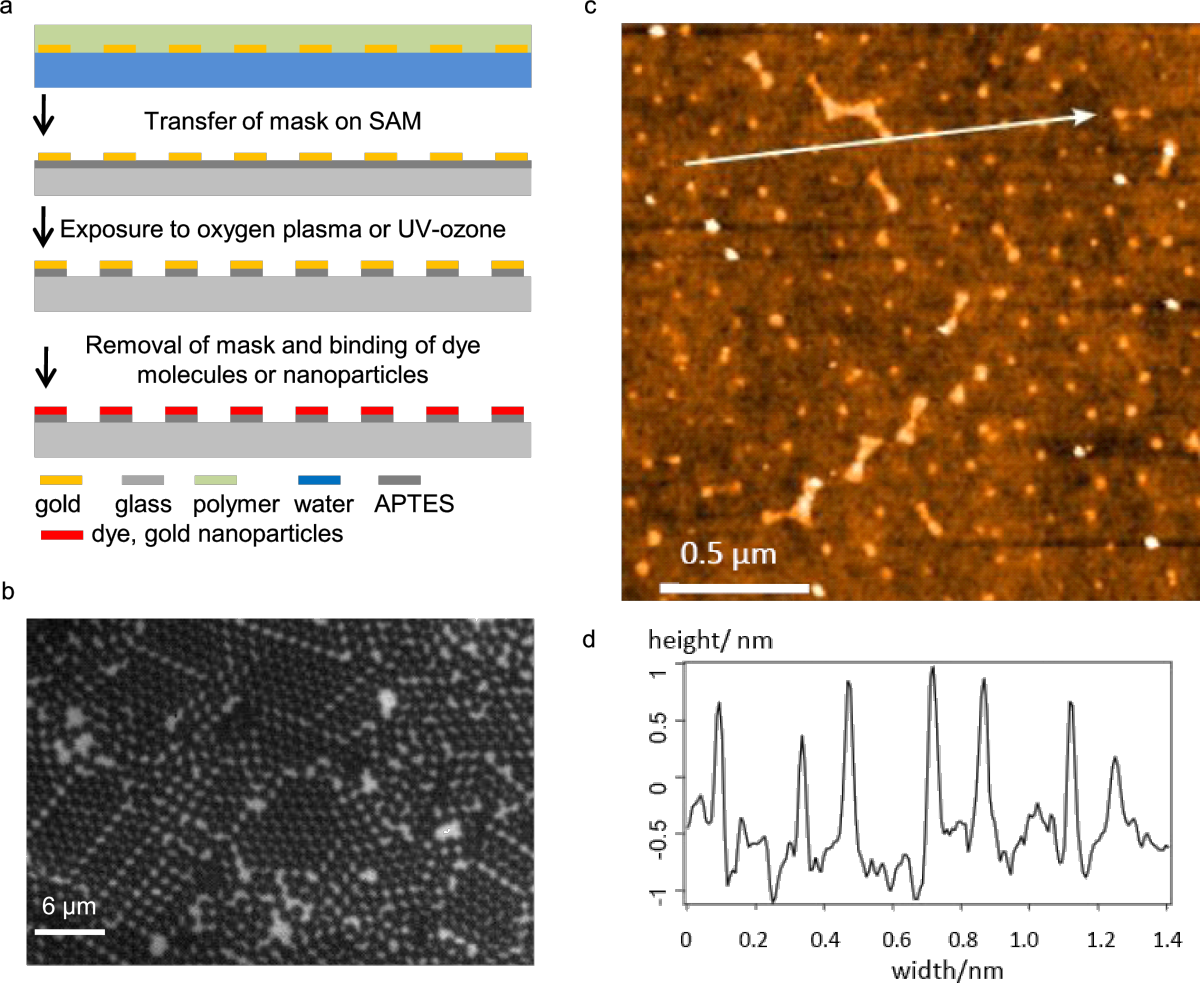
Oxygen-plasma induced chemical nanostructuring (process 2). (a) Schematic representation of the major preparation steps to form a chemically functionalized nanopattern. (b) Fluorescence micrograph of an APTES SAM nanopattern obtained by process 2 using mask 2 produced from a projection pattern of 1.2 µm latex beads after covalent binding of fluorescein molecules to the amino groups of the APTES. (c) Topography recorded by an atomic force microscope of an APTES SAM nanopattern obtained by the same procedure using a mask produced from a projection pattern of 0.22 µm latex beads after binding of negatively charged 1.4 nm gold nanoparticles to the positively charged amino groups of APTES. (d) Profile along the line indicated in (c).

In order to support the role of the intimate contact between the metal mask and the fluorescein molecules a fluorescence image of the mask in contact with the monolayer was acquired. Figure 2d shows 1.2 µm projection patterns and the remaining fluorescence of the monolayer in the unprotected areas. The intimate contact regions in form of triangular metal islands appear dark (Figure 2b) due to quenching of the fluorescence at sites of contact between metal and dye monolayer before bleaching the dye. (The latter experiments were performed utilizing mask 2 instead of mask 1 since the illumination of the gold film of mask 1 resulted in reflections which were not sufficiently suppressed in our epi-fluorescence microscope such that the fluorescence quenching at sites of metal contact could not be recognized). For the processes 2 and 3, oxygen plasma and UV–ozone nanopatterning, as outlined in Figure 3a, and described in more detail in the Experimental section, the embedded mask 2 was transferred from the glass substrate onto a SAM (e.g., an APTES SAM). After dissolving of the supporting polymer film, the sandwich of mask and SAM was exposed in process 2 to the oxygen plasma. After exposure, the mask was removed mechanically.

During treatment with the oxygen plasma it is assumed that the APTES self-assembled monolayer is locally degraded in the non-protected areas, which are not covered by the mask 2. This degradation is not directly visible and thus a fluorescent label was introduced, which selectively attaches to the preserved amine functionalities.

Figure 3b shows a fluorescence micrograph of the APTES SAM pattern after chemical binding of the fluorescein tags to the amino groups of the APTES SAM. A fluorescence image of the 1.2 µm nanopattern was found (Figure 3b). Clearly the hexagonally aligned triangular fluorescence pattern is observed. Additional experiments included the labeling of the fabricated chemical nanopatterns with nanoparticles, which was performed to increase the topographical contrast for detection by means of AFM. By binding 1.4 nm negatively charged gold nanoparticles to the positively charged APTES SAM nanopattern it could be demonstrated that the pattern transfer was successful even for 0.22 µm masks (Figure 3c). The topographic features reveal a uniform height of approximately 1.5 nm of the bound particles. However with the AFM we were not able to detect the specific binding of fluorescine tags to the triangular spots of a size of the order of 30 nm which could be detected in fluorescence micrographs at diffraction limited resolution for the 1.2 µm patterns. Also longer times of exposure to the oxygen plasma of the order of 1 min led to a strong deterioration of the fluorescence of the tagged 1.2 µm APTES SAM nanopatterns such that only a coarse fluorescent structure could be detected whereas the triangular patches disappeared. These findings indicate, that the small features of the gold mask do not lead to a complete inhibition of the destruction of the amino groups by the oxygen plasma. Chemically functional APTES SAM nanostructures were thus formed by the etching with oxygen plasma only at a resolution of the order of 200 nm.

**Figure 4:**
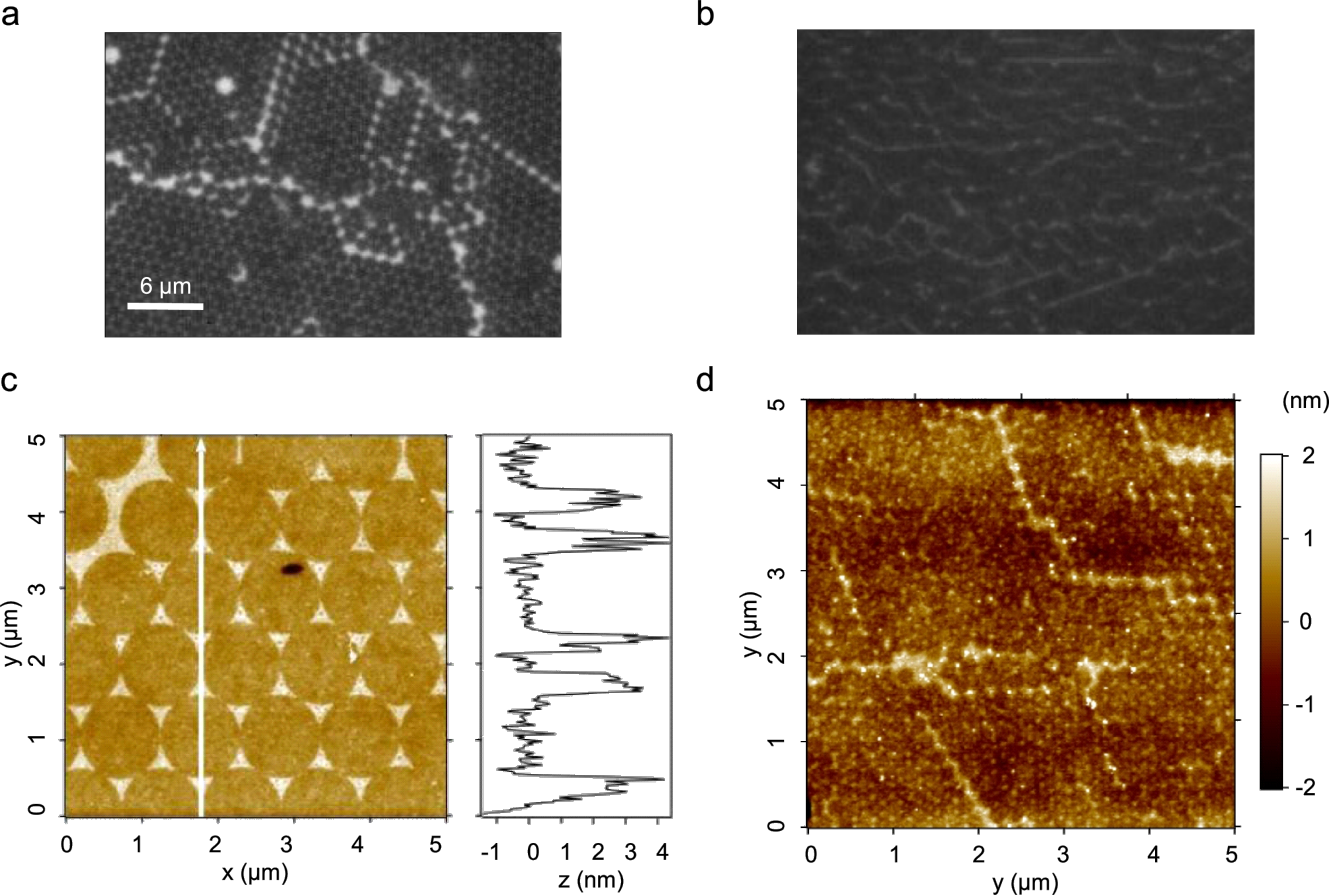
Hybrid UV-light and ozone near-field chemical nanostructuring of an ATPES-SAM (process 3). Fluorescence micrographs of nanopatterns based on masks obtained from projection pattern of (a) 1.2 µm latex beads and of (b) 220 nm latex beads after staining of the chemically functionalized areas with fluorescein molecules. Optically only the larger hexagonal nanopattern can be resolved, whereas the fluorescence image of the 220 nm pattern is characterized by defect structures. (c) AFM micrograph of 1.4 nm negatively charged nanoparticles site-selectively bound to triangular shaped APTES-functionalized nanostructures reproduced the detailed lateral structure of the applied mask. (d) Topography image of the same fluorescein stained sample as shown in (b).

Alternatively, a UV–ozone treatment is applied in process 3. After removal of the gold mask, the resulting APTES SAM nanopattern is decorated by binding of negatively charged gold nanoparticles or fluorescent molecules to the amino groups of APTES. The results of nanopatterns fabricated by UV-light and ozone exposure in the presence of mask 2 are summarized in Figure 4. APTES SAM nanopatterns were formed with 1.2 µm as well as 0.22 µm masks. Fluorescent micrographs of 1.2 µm and of 0.22 µm nanopatterns after binding of fluorescein tags are shown in Figure 4a and Figure 4b, respectively.

The 1.2 µm structure is clearly resolved in the fluorescence image, but due to the diffraction limit the 0.22 µm pattern cannot be resolved. Only the defect structures of the periodic nanopatterns are recognized. In addition to the staining of the chemical nanostructures with fluorescein molecules, the nanopatterns could be visualized by AFM topography maps after decoration of the formed patterns with 1.4 nm Au nanoparticles (Figure 4c). The corresponding topographic image of a 1.2 µm APTES SAM pattern reveals periodic triangular shaped structures of 3 nm height in most parts of the image (Figure 4c). This indicates that in this case a double layer of the negatively charged gold nanoparticles might be adsorbed. Unlike in the case of the specific chemical binding of the fluorescein tags, more or less than a monolayer can be bound to the positively charged nanopattern due to charge compensation, depending on the surface charges of the nanopattern and of the nanoparticles which may alter their properties under different conditions of the sample preparation.

For the 0.22 µm nanopattern, which cannot be identified in the fluorescence images, the topography images taken on the same fluorescein-stained APTES SAMs clearly reveal the typical periodicity of the expected pattern (Figure 4d). The fact that even the weak topographic features of the fluorescein-labeled APTES-SAM nanopattern can be detected with the AFM at high lateral resolution indicates, that the masks lead to a very strong and highly localized inhibition of the photochemical process induced by the UV–ozone treatment.

## Discussion

The methods described here have great potential to generate nanopatterns as templates for the selective binding of molecules or nanoparticles with high spatial resolution. The high resolution potential can be related to the inhibitory function of the mask as described in detail for the near-field photochemical process [24]. If the inhibitory function of the metal mask is very strong, a long exposure time of the sample leads to a complete reaction at the sites where there is no contact to the metal mask, whereas the reaction does not take place at the sites of contact. Thus, a close contact and the inhibitory function of the masks are the prerequisite for obtaining a high resolution in these near-field processes. A thin reactive layer, such as a SAM, is one prerequisite to obtain a close contact. With the near-field photochemical process, a long exposure of about 1 h resulted in a pattern of domains of bleached and unbleached dye. This process was identified as a very suitable high resolution structuring tool. It is desirable to exploit the chemical nanopatterns for a selective binding of molecules and nanoparticles. It is not obvious how to realize such a selectivity on the basis of a photochemical process that uses light in the visible spectral range. This limits the overall applicability of the near-field photochemical process using visible light. UV photochemical processes and chemical processes are more suitable to generate chemical nanostructures with selective binding properties. Utilizing oxygen-plasma treatment as a chemical process preferentially results in the destruction of the unprotected surface areas but the protective properties of the mask were not sufficient to obtain well-defined chemically functional nanostructured SAMs at a resolution below 200 nm. A more pronounced inhibitory function of the gold mask for the hybrid chemical and photochemical UV–ozone treatment than for oxygen-plasma treatment may be the reason for the better quality of the obtained APTES SAM nanopatterns as evidenced by the detection of even the topographic features at a lateral resolution of 30 nm formed by the fluorescein-labeled 0.22 µm pattern. The use of mechanically rather stable, chemically bound SAMs as substrates for the processes [27–28] facilitates the procedures for contacting and removal of the masks. Here, contacting the mask by evaporation of a drop of water between the SAM and the flexible mask and dissolution of the backing plastic film leads to a good contact between the mask and the SAM. Removal of the mask either by Scotch tape or mechanical wiping with a tissue was possible due to the choice of gold as metal for the mask which does not adhere strongly to the substrates and to the mechanically stable SAMs.

Further work will be necessary to develop a more reliable and better defined process for the removal of the mask. Although the potential of CL was not yet fully exploited to test the resolution potential of the fabrication methods, other methods of bottom-up fabrication of the metal masks are desirable for this purpose. For the fabrication of metal masks as used here the block-copolymer micelle nanolithography [18], the silver decoration technique or other approaches using biological templates to create films of ordered islands of silver or gold may be a candidates to further scale down the masks to dimensions below 10 nm [29–30].

## Conclusion

Three realizations of near-field photochemical and radiation-induced chemical fabrication processes of SAM nanopatterns were explored: 1) a photochemical process, in which chemical nanopatterning is achieved by selective photochemical bleaching of a monolayer of dye molecules chemically bound to an APTES SAM; 2) a chemical process by oxygen-plasma etching as well as 3) a combined UV-photochemical and ozone-induced chemical process for SAMs of organosilanes. All approaches employ a sandwich configuration of a surface-supported SAM and a lithographic mask in form of gold nanostructures fabricated by colloidal sphere lithography (CL), which is either exposed to visible light, oxygen plasma or an UV–ozone atmosphere. The gold mask has the function to inhibit the photochemical reactions of the SAM by highly localized near-field interactions between mask and SAM and to inhibit the radiation-induced chemical reactions by casting a highly localized shadow. Removal of the gold mask reveals the SAM nanopattern. As a result of all three processes nanopatterns of SAMs were obtained with a resolution of about 30 nm. The formation of nanopatterns by local bleaching was demonstrated with the near-field photochemical process but these patterns could not be used for the selective attachment of molecules or nanoparticles. Patterning by oxygen plasma led to a chemically functional SAM nanopattern of limited resolution of the order of 200 nm. Only the combined UV near-field photochemical and ozone-induced chemical process led to functional nanopatterns of a chemically reactive SAM which could be used for the selective attachment of molecules at a resolution of 30 nm.

## Experimental

### Mask fabrication and mask transfer

The fabrication of mask 1 involves the following steps: A gold film is prepared through thermal evaporation of gold onto a cover glass. Then, a thin film of polystyrene is deposited onto the gold film by spin coating of a 2% solution of polystyrene (MW 100000) in toluene at 1500 rpm. The sandwich layer of the gold film and the polystyrene film is cut with a diamond marker into squares of about 2–3 mm. By dipping the coated cover glass slowly at an oblique angle into a water trough, the films are separated from the glass substrate by a thin film of water penetrating the glass–gold interface thus leaving the gold film floating on the water surface. The film is transferred onto the glass-supported projection pattern (Figure 1b). For that purpose, single squares are trapped in a small metal loop. Lifting the loop through the water surface removes the squares floating on a drop of water, which is suspended by the loop. The loop with the water drop is then deposited onto the gold projection pattern. The water is partially removed by touching the side of the loop with a piece of filter paper and the loop is then removed leaving the sandwich film on the projection pattern. The sample is then exposed to a vacuum of 10^−1^ mbar to remove the remaining water and to obtain an intimate contact between the gold film and the projection pattern over the whole sample area except for a few unavoidable wrinkles, which are formed in this process. Mask 1 can then be transferred onto a water surface in the same way as the gold film as described above. The contact between the gold projection pattern and the gold film in mask 1 is strong enough that the integrity of mask 1 is not affected by the transfer process. In order to be used in process 1 mask 1 is picked up from the water surface and transferred onto a fluorescein SAM monolayer by the same transfer process as described above.

Mask 2 for the processes 2 and 3 consists of 30 nm thick gold projection patterns obtained by CL of 1.2 µm or 0.22 µm latex beads. The gold projection patterns are prepared on a glass substrate. Instead of backing this master with a thin gold film the formed metal structure is subsequently embedded in a flexible polymer matrix by spin coating of polystyrene dissolved in toluene as described above. The 1 mm^2^ patches of the embedded masks are floated onto a clean water surface in the same way as described above for the gold film, leading to a lift-off of the masks. The embedded mask 2 floating on the water surface can be simply transferred in the next step onto different functionalized solid surfaces. Before exposure to process 2 or 3 the supporting polystyrene film is removed by dissolution in toluene.

### Functionalized SAMs

Commercially available APTES SAMs on cover glass slides of a thickness of 0.18 mm as obtained from Polyan GmbH, Germany, were used as a substrate for the three individual processes. They are directly used as substrates for structuring processes utilizing oxygen plasma or UV–ozone treatment. For the contact imaging energy transfer lithography approach the amine reactive fluorescent molecules fluorescein-5-EX/succinimidylester as obtained from Molecular Probes of Life Technologies, U.S.A. were chemically bound to the amino groups of the APTES SAM. For the reaction 250 µg of the dye was dissolved in 450 µL dimethylsulfoxide to which 50 µL triethylamine was added. A drop of 100 µL was spread on the APTES SAM cover glass and left to react for 2 h. After the reaction the glasses were washed with DMSO and water in an ultrasonic bath.

### Exposure conditions

In process 1 an area of about 1 mm^2^ of the sandwich structure was exposed to light of a 75 W high pressure Xenon arc lamp for 1 h in an epi-fluorescence microscope by using an objective lens of a NA of 0.5. According to the absorption peak of the fluorescein dye at around 496 nm a bandpass filter around 480 nm was used to define the spectral region of the excitation source. After exposure and removal of the mask the sample was cleaned in an ultrasonic water bath and thoroughly rinsed with water.

For process 2 a sandwich of mask 2 and an APTES SAM was exposed for 10 s to an oxygen plasma at an oxygen pressure of 0.1 mbar and a power of 150 W (Model 100 Tepla AG, Germany). Afterwards the mask was removed by mechanical wiping of the substrate with a tissue soaked in acetone. For process 3 the same sandwich structure as used for process 2 was exposed for 5 min to ultraviolet light (184.9 nm and 253.7 nm) and ozone in an UVO cleaner (144-AX Jelight Company Inc. U.S.A.). After exposure the mask was removed in the way mentioned above.

### Labeling with fluorescein tags and gold nanoparticles

Fluorescein tags were bound to the APTES SAM nanopatterns following the same reaction scheme as utilized for the fabrication of the fluorescein functionalized substrates and described above. For binding of gold nanoparticles to such APTES SAM nanopatterns, a 10^−4^ M solution of negatively charged gold nanoparticles of an average size of 1.4 nm as obtained from Nanoprobes Inc., U.S.A was spread on the APTES SAM nanopatterns and was left to react for 1 h in an atmosphere which was saturated with the solvent. After the reaction the cover glass was rinsed with water.

## References

[1] Falconnet D, Pasqui D, Park S, Eckert R, Schift H, Gobrecht J, Barbucci R, Textor M (2004). Nano Lett.

[2] Mendes P M, Yeung C L, Preece J A (2007). Nanoscale Res Lett.

[3] Christman K L, Broyer R M, Schopf E, Kolodziej C M, Chen Y, Maynard H D (2010). Langmuir.

[4] Park S, Frey W (2011). Langmuir.

[5] Becer C R, Haensch C, Hoeppener S, Schubert U S (2007). Small.

[6] Kim S O, Kim B H, Meng D, Shin D O, Koo C M, Solak H H, Wang Q (2007). Adv Mater.

[7] Lee J Y, Lee J, Jang Y J, Lee J, Jang Y H, Kochuveedu S T, Park C, Kim D H (2011). Chem Commun.

[8] Hoeppener S, Schubert U S (2005). Small.

[9] Kang S-W, Banerjee D, Kaul A B, Megerian K G (2010). Scanning.

[10] Lau K H A, Bang J, Hawker C J, Kim D H, Knoll W (2009). Biomacromolecules.

[11] Lim J L, Danohue H J (2007). Tissue Eng.

[12] Aizenberg J (2004). Adv Mater.

[13] Ruiz R, Kang H, Detcheverry F A, Dobisz E, Kercher D S, Albrecht T R, de Pablo J J, Nealey P F (2008). Science.

[14] Arnold M, Hirschfeld-Warneken V C, Lohmüller T, Heil P, Blümmel J, Cavalcanti-Adam E A, López-Garcia M, Walther P, Kessler H, Geiger B (2008). Nano Lett.

[15] Zeira A, Chowdhury D, Maoz R, Sagiv J (2008). ACS Nano.

[16] Beyer A, Godt A, Amin I, Nottbohm C T, Schmidt C, Zhao J, Gölzhäuser A (2008). Phys Chem Chem Phys.

[17] Haryono A, Binder W H (2006). Small.

[18] Lohmüller T, Aydin D, Schwieder M, Marhard C, Louban I, Pacholski C, Spatz J P (2011). Biointerphases.

[19] Huang C, Moosmann M, Jin J, Heiler T, Walheim S, Schimmel T (2012). Beilstein J Nanotechnol.

[20] Fischer U C, Zingsheim H P (1981). J Vac Sci Technol.

[21] Deckman H W, Dunsmuir J H (1982). Appl Phys Lett.

[22] Yang S-M, Jang S G, Choi D-G, Kim S O, Yu H K (2006). Small.

[23] Herzer N, Hoeppener S, Schubert U S, Fuchs H, Fischer U C (2008). Adv Mater.

[24] Fischer U C, Zingsheim H P (1982). Appl Phys Lett.

[25] Kuhn H (1970). J Chem Phys.

[26] Herzer N, Hoeppener S, Schubert U S (2010). Chem Commun.

[27] Herzer N, Eckardt R, Hoeppener S, Schubert U S (2009). Adv Funct Mater.

[28] Herzer N, Haensch C, Hoeppener S, Schubert U S (2010). Langmuir.

[29] Sleytr U B, Messner P, Pum D, Sára M (1999). Angew Chem, Int Ed.

[30] Neugebauer D-C, Zingsheim H P (1978). J Mol Biol.

